# Suppression of anoikis in human intestinal epithelial cells: differentiation state-selective roles of α2β1, α3β1, α5β1, and α6β4 integrins

**DOI:** 10.1186/1471-2121-14-53

**Published:** 2013-12-01

**Authors:** Marco Beauséjour, Sonya Thibodeau, Marie-Josée Demers, Véronique Bouchard, Rémy Gauthier, Jean-François Beaulieu, Pierre H Vachon

**Affiliations:** 1 Département d’anatomie et de biologie cellulaire, Faculté de médecine et des sciences de la santé, Université de Sherbrooke, J1H5N4 Sherbrooke, Québec, Canada

**Keywords:** Anoikis, Integrin, Signaling

## Abstract

**Background:**

Regulation of anoikis in human intestinal epithelial cells (IECs) implicates differentiation state-specific mechanisms. Human IECs express distinct repertoires of integrins according to their state of differentiation. Therefore, we investigated whether α2β1, α3β1, α5β1, and α6β4 integrins perform differentiation state-specific roles in the suppression of IEC anoikis.

**Results:**

Human (HIEC, Caco-2/15) IECs were exposed to specific antibodies that block the binding activity of integrin subunits (α2, α3, α5, α6, β1 or β4) to verify whether or not their inhibition induced anoikis. The knockdown of α6 was also performed by shRNA. Additionally, apoptosis/anoikis was induced by pharmacological inhibition of Fak (PF573228) or Src (PP2). Anoikis/apoptosis was assayed by DNA laddering, ISEL, and/or caspase activity (CASP-8, -9, or -3). Activation levels of Fak and Src, as well as functional Fak-Src interactions, were also assessed. We report herein that differentiated IECs exhibit a greater sensitivity to anoikis than undifferentiated ones. This involves an earlier onset of anoikis when kept in suspension, as well as significantly greater contributions from β1 and β4 integrins in the suppression of anoikis in differentiated cells, and functional distinctions between β1 and β4 integrins in engaging both Fak and Src, or Src only, respectively. Likewise, Fak performs significantly greater contributions in the suppression of anoikis in differentiated cells. Additionally, we show that α2β1 and α5β1 suppress anoikis in undifferentiated cells, whereas α3β1 does so in differentiated ones. Furthermore, we provide evidence that α6β4 contributes to the suppression of anoikis in a primarily α6 subunit-dependent manner in undifferentiated cells, whereas this same integrin in differentiated cells performs significantly greater contributions in anoikis suppression than its undifferentiated state-counterpart, in addition to doing so through a dependence on both of its subunits.

**Conclusions:**

Our findings indicate that the suppression of human IEC anoikis implicates differentiation state-selective repertoires of integrins, which in turn results into distinctions in anoikis regulation, and sensitivity, between undifferentiated and differentiated IECs. These data further the functional understanding of the concept that the suppression of anoikis is subjected to cell differentiation state-selective mechanisms.

## Background

Cell-extracellular matrix (ECM) interactions play crucial roles in the regulation of the various known cellular processes [1-4]. The biological functions attributed to cell-ECM interactions are mediated primarily by heterodimeric (αβ) transmembrane receptors of the integrin family [4-8]. So far, 18 α subunits and 8 β subunits have been identified in humans, with α subunits non-covalently associating with β subunits, consequently forming 24 distinct heterodimeric (αβ) receptors with differing ligand specificities [4-9]. Some α and β subunits can undergo post-transcriptional alternative mRNA splicing, or post-translational proteolytic processing [4-9]. This largely results in variants with alterations in their cytoplasmic tails, thus adding further versatility to their roles and functions [4-9]. It is those integrins that have the β1 subunit in common which constitute the majority of receptors for ECM components [4-9]. Also of this group is the α6β4 integrin, which is expressed exclusively in epithelial cells [4,6].

The binding of an integrin to its ECM ligand generates a vast range of transduction signals which affect cell behavior, cell shape, and gene expression [2,4,6,8-10]. To this effect, signaling by β1 integrins owes largely to the recruitment and activation of the tyrosine kinase Fak. In turn, Fak typically recruits and activates the tyrosine kinase Src [1,2,4,8-12]. Conversely, the α6β4 integrin engages Src, but not Fak [4,6,12,13]. Regardless, integrin-mediated signal transduction involves the downstream engagement of a plethora of pathways, largely due to the formation of diverse signaling cassettes through the recruitment by Fak, and/or Src, of an increasing array of macromolecules [1,2,4,6,8-13]. In this respect, it is established that a given repertoire of expressed integrins not only engenders distinct signals for a specific cell type, but also exerts a differential modulation of cell processes within the same tissue [1-4,6,8-13].

Caspase-dependent apoptosis constitutes a finely regulated process which performs crucial functions in tissue development and homeostasis [1,2,4,14,15]. It is now well understood that normal cells are intrinsically wired by default to undergo apoptosis and, consequently, require the input of signals in order to maintain the process in a suppressed mode when not warranted [1,2,4,14,15]. One of the critical biological roles performed by cell-ECM interactions is the maintenance of cell survival [1,2,4,6,9,11-13,16,17]. To this effect, normal cells undergo caspase-dependent apoptosis through a process termed *anoikis* (a.k.a. “detachment-induced apoptosis”, or “integrin-mediated death”) whenever a disruption, or loss, of integrin-mediated anchorage occurs [1,2,4,6,9,11-13,16-20]. Indeed, integrin signaling, largely via the activation of Fak and/or Src, leads to the engagement of numerous pathways that promote cell survival and the suppression of anoikis [1,2,4,6,9-13,16-20].

The main distinction between apoptosis and anoikis lies with the activation of CASP-8 as initiator caspase in the latter [2,4,18-21], although such activation ultimately leads to the activation of the common apoptotic initiator CASP-9, in order to render the process irreversible [2,4,18-20]. Like apoptosis, anoikis performs important functions during organogenesis, as well as in tissue maintenance and renewal [1,2,4,6,9,11-13,16,17],[19,20]. In this respect, it is now recognized that normal cells are endowed with a default anchorage-dependent surveillance system, which is responsible for upholding the correct position of cells within their respective tissues, and thereby sentencing to death-by-anoikis any cell that would stray from its assigned position – by either interacting with an inappropriately composed ECM, or by losing anchorage altogether [1,2,4,9,16,17,19,20].

The intestinal epithelium is a useful physiological system for understanding the functional connections between integrin-mediated cell-ECM interactions and the cell state [22-26]. The continuous renewal of this simple columnar epithelium occurs along a well-defined unit, the crypt-villus axis. This unit consists generally in two cell populations: the proliferative, immature cells of the crypt, and the differentiated cells of the villus [22-28]. As part of the dynamic process of intestinal epithelial cell (IEC) renewal, obsolete IECs typically enter anoikis upon reaching the apex of the villi, as a means of exfoliation [23,24,27,28]. For their part, crypt cells occasionally undergo apoptosis in order to remove daughter cells that are damaged or defective [23,24,27,28]. Such apparent contrast of destiny between undifferentiated and differentiated IECs has been shown to implicate differentiation state-related distinctions in the regulation of cell survival, apoptosis, and anoikis [4,23,24,29-37]. Incidentally, crypt and villus IECs express differential profiles of integrins as they interact with specific ECM components, which are likewise deposited differentially, along the crypt-villus axis [4,22-24,26,27]. Hence, the question remains open as to whether such differentiation state-specific repertoires of integrins contribute distinctively in the regulation of IEC anoikis.

In this study, we investigated the roles of the α2β1, α3β1, α5β1 and α6β4 integrins in the suppression of anoikis in undifferentiated (HIEC, Caco-2/15-2PC) and differentiated (Caco-2/15 30PC) human IECs, including with regards to their contributions in the activation of Fak and/or Src. Herein, we show that differentiated IECs exhibit a greater sensitivity to anoikis than undifferentiated ones, when kept in suspension. To this effect, we find that β1 and β4 integrin subunit-containing integrins, as well as Fak, perform significantly greater contributions in the suppression of anoikis in differentiated cells. Additionally, we show that the α2β1 and α5β1 integrins suppress anoikis in undifferentiated cells only, whereas α3β1 does so exclusively in differentiated ones. Furthermore, α6β4 performs significantly greater contributions in the suppression of anoikis, in differentiated cells. We also provide evidence that α6β4 contributes to the suppression of anoikis in a primarily α6 subunit-dependent manner in undifferentiated cells, whereas in differentiated cells, this same integrin does so through a dependence on both of its subunits. Taken together, our results indicate that the suppression of human IEC anoikis implicates differentiation state-selective repertoires of integrins, which in turn results into distinctions in anoikis regulation, and sensitivity, between undifferentiated and differentiated IECs. Lastly, these findings further the functional understanding of the concept that cell survival, and the suppression of anoikis, are subjected to cell differentiation state-selective mechanisms.

## Results

### Human IECs display a distinct sensitivity to anoikis according to their state of differentiation

**Figure 1:**
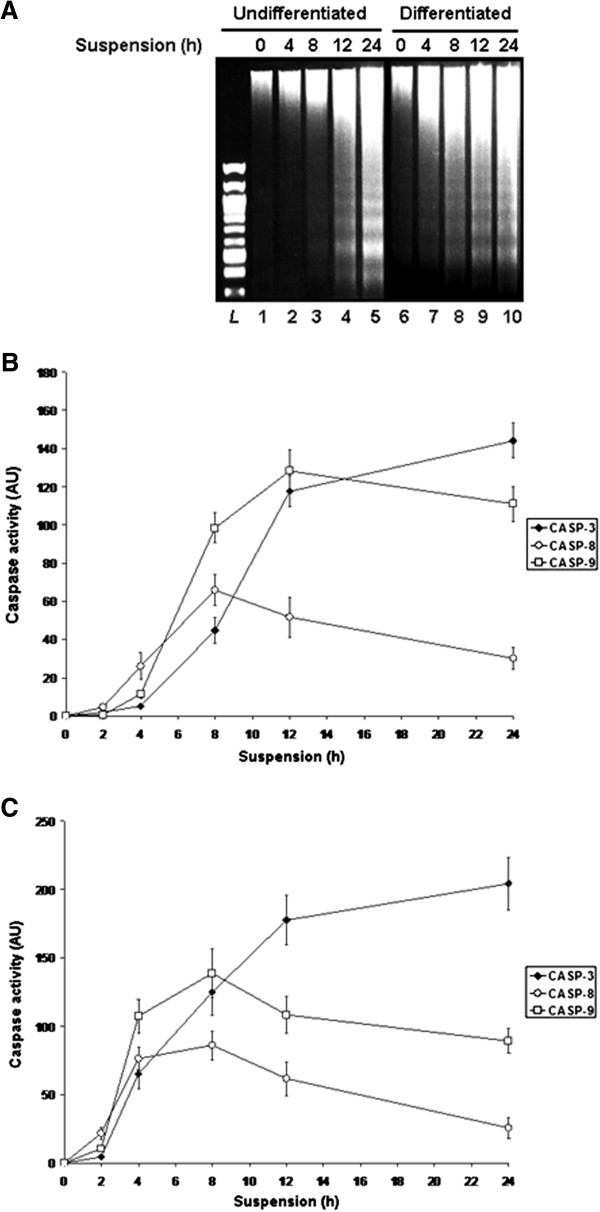
**Human IECs exhibit differentiation state-associated distinctions in anoikis sensitivity. A.** Representative (n ≥ 3) time-course kinetics of CAD-mediated DNA laddering from -2PC (*Undifferentiated*; lanes 1-5) and 30PC (*Differentiated*; lanes 6-10) Caco-2/15 cells maintained 0-24 h in suspension, serum-free. *L*, 100-bp DNA size markers. **B.** Time-course kinetics (n ≥ 3) of caspase activity for CASP-3 (filled diamonds), CASP-8 (open circles) and CASP-9 (open squares), from -2PC Caco-2/15 cells maintained as in **(*A*)**. **C.** Same as in **(*B*)**, except that CASP-8, -9 and -3 activities were assessed from 30PC Caco-2/15 cells maintained as in **(*A*)**. **A-B.** Results obtained with HIEC cells were highly similar to those shown here for -2PC Caco-2/15 cells.

We first established a time-course appearance of caspase-activated DNAse (CAD)-mediated DNA laddering, in both undifferentiated (HIEC and/or Caco-2/15 -2PC) and differentiated (Caco-2/15 30PC) IECs, maintained 0-24 h in suspension. In undifferentiated cells, DNA laddering was weakly discernible around 8 h, in order to increase in intensity to a maximum after 24 h (Figure 1A), as we reported previously [30,32,33]. While similar kinetics of DNA laddering were observed in differentiated cells, we found that internucleosomal DNA fragmentation became weakly discernible round the 4 h time-point, instead of the 8 h one noted for their undifferentiated counterparts (Figure 1A), as we previously reported [30,32,33].

We then established the concomitant time-course kinetics of the specific activities of the initiator caspases CASP-8 and -9, as well as that of the executioner caspase CASP-3 (which is responsible for the activation of CAD [2,4,19,20]). In undifferentiated IECs, CASP-8 activity was weakly detectable around 2 h, thereafter peaking around 8 h (Figure 1B). CASP-9 activity was weakly detectable around 4 h and peaked around 12 h (Figure 1B), indicating that its activation/activity followed that of CASP-8, as expected [2,4,18-21,24]. In turn, CASP-3 activity was likewise weakly detectable around 4 h, increasing thereafter to a maximum around the 24 h time-point (Figure 1B). Overall, the kinetics of CASP-8, -9 and -3 activities paralleled that of the appearance of internucleosomal DNA fragmentation (Figure 1A-B). Of particular interest is the 8 h time-point, where all three caspases began exhibiting strong-to-high activities, thus coinciding with the emergence of discernible DNA laddering (Figure 1A-B). Although similar kinetics of CASP-8, -9 and -3 activities were observed in differentiated IECs, which likewise paralleled that of the appearance of DNA laddering in these cells (Figure 1A, C), we found that it was instead at the 4 h time-point where all three caspases began exhibiting strong-to-high activities (Figure 1C). Again, this coincided with the emergence of discernible internucleosomal DNA fragmentation (Figure 1A).

**Figure 2:**
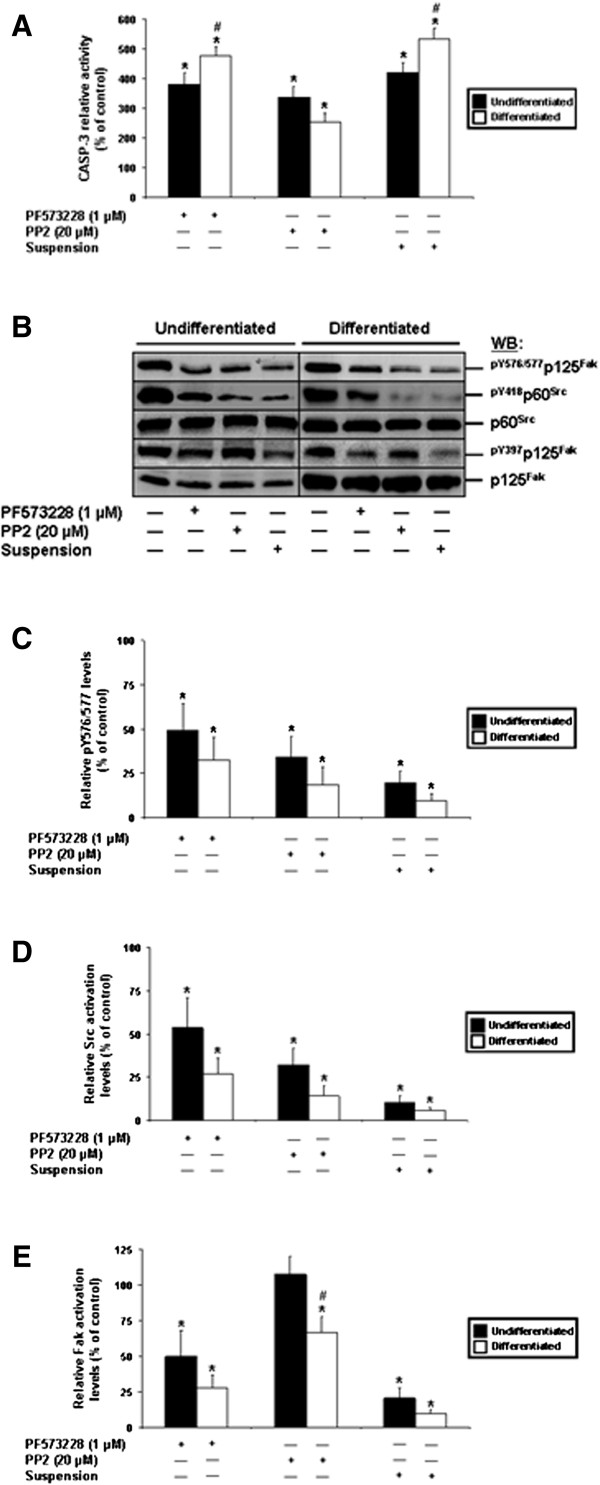
**Roles of Fak and Src in the suppression of anoikis in human IECs. A.** HIEC (*Undifferentiated*; filled columns) and 30PC Caco-2/15 (*Differentiated*; open columns) adhering cell cultures were maintained 24 h serum-free (control) with PF573228 (Fak inhibitor) or PP2 (Src inhibitor), or maintained instead in suspension. CASP-3 relative activity was then established, by comparison to controls. Results obtained with -2PC Caco-2/15 cells were comparable to those shown here for HIEC cells. **B.** Representative (n ≥ 5) WB analyses of the activation of Src and Fak, and verifications of Fak-Src interactions, from -2PC (*Undifferentiated*) and 30PC (*Differentiated*) Caco-2/15 cell cultures maintained as in **(*A*)**. Specific antibodies for ^pY576/577^p125^Fak^, ^pY418^p60^Src^ and ^pY397^p125^Fak^, as well as for respective total protein forms, were used. **C-E.** -2PC (*Undifferentiated*; filled columns) and 30PC (*Differentiated*; open columns) Caco-2/15 cells were maintained and processed as in **(*A-B*)**, except that the relative pY576/577 levels of Fak **(*C*)**, as well as the relative activation levels of Src **(*D*)** and Fak **(*E*)**, were established in comparison to controls. **B-E.** Results obtained with HIEC cells were highly similar to those shown here for -2PC Caco-2/15 cells. **A, C-E.** Statistically significant (0.0001 ≤ *P* ≤ 0.001; n ≥ 5) differences between treated and control cultures are indicated by (*). Statistically significant (0.0005 ≤ *P* ≤ 0.005; n ≥ 5) differences between differentiated and undifferentiated IECs are indicated by (#).

We have previously shown that the integrin-mediated suppression of anoikis in human IECs engages Fak and Src [29-33]. Hence, we analyzed the impacts of the pharmacological inhibition of Fak and Src in both undifferentiated and differentiated IECs, in relation to the maintenance of these cells in suspension. Controls consisted of adhering, non-treated cell cultures. As shown in Figure 2A, the inhibition of Fak, Src, or the maintenance in suspension, induced significant anoikis in both undifferentiated and differentiated cells. Interestingly, the inhibition of Fak, as well as the maintenance in suspension, induced anoikis at significantly greater levels in differentiated IECs. However, the inhibition of Src induced apoptosis/anoikis without significant differences between undifferentiated IECs and their differentiated counterparts (Figure 2A).

The relative activation levels of Fak and Src were then analyzed, in both control and treated cultures, in order to validate the efficiency of our treatments. Additionally, functional Fak-Src interactions were assessed by analyzing the relative phosphorylation levels of the Y576/577 residues of Fak, enacted by Src [4,11,32-35]. As expected [32-35], the inhibition of Fak in both undifferentiated and differentiated IECs caused a significant down-activation of Fak itself and of Src, along with a significant decrease in Fak-Src interactions, as when cells were kept in suspension (Figure 2B-E). In the same vein, the inhibition of Src in both undifferentiated and differentiated IECs resulted in its own down-activation and a significant decrease in Fak-Src interactions, again as when cells were kept in suspension (Figure 2B-D). Surprisingly, while the inhibition of Src had no effect on the activation of Fak in undifferentiated cells, it did result in a significant down-activation of Fak in differentiated ones (Figure 2B, E).

Therefore, these data altogether establish firmly that differentiated human IECs exhibit a sensitivity to anoikis that is distinct from their undifferentiated counterparts. Additionally, these results not only show that such differentiation state-distinctions are associated with specific contributions from Fak and Src in suppressing anoikis, but furthermore suggest a differentiation state-selective crosstalk between Fak and Src, with regards to their respective activation.

### Differentiation state-distinct contributions of β1 and β4 integrins in the suppression of anoikis in human IECs

To further understand mechanistically the differentiation state-related distinctions in the regulation of anoikis in human IECs, cells were exposed to specific mouse monoclonal antibodies directed to the extracellular domain of the β1 (mAb P4C10) or β4 (mAb 3E1) integrin subunits, in order to inhibit their binding activity. Controls constituted of cell cultures exposed to mouse IgG’s (control for blocking mAbs), or to no antibodies/IgG’s (basal control). It must be noted here that human IECs express the integrin subunits β1 and β4, regardless of the state of differentiation [22,23,26,38]. However, undifferentiated/crypt human IECs, but not differentiated/villus ones, express a co-translational proteolysis-processed β4 variant that lacks a small COOH-terminal fragment in its cytoplasmic domain (dubbed “β4^ctd-^”) [22,26,38].

**Figure 3:**
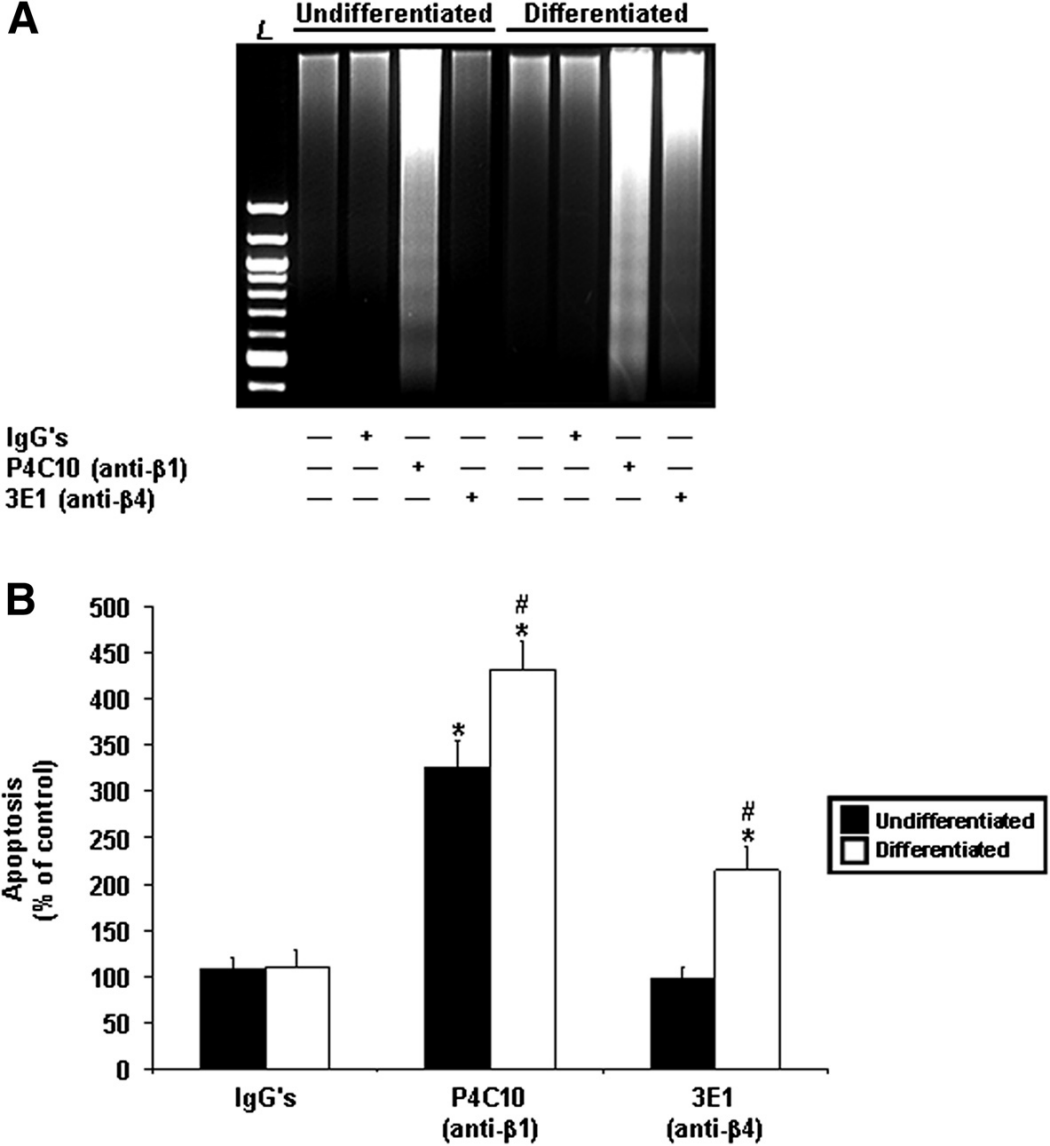
**Differentiation state-distinct contributions of β1 and β4 integrins in the suppression of human IEC anoikis. A.** Representative (n ≥ 3) CAD-mediated DNA laddering assays from HIEC (*Undifferentiated*) and 30PC Caco-2/15 (*Differentiated*) adhering cell cultures, maintained 24 h serum-free (control) with mouse IgG’s, P4C10 (β1 binding activity-blocking mAb), or 3E1 (β4 binding activity-blocking mAb). *L*, 100-bp DNA size markers. **B.** HIEC (*Undifferentiated*; filled columns) and 30PC Caco-2/15 (*Differentiated*; open columns) cell cultures were maintained as in **(*A*)**, except that ISEL assays were performed and compared to controls. Statistically significant (0.0001 ≤ *P* ≤ 0.001; n ≥ 6) differences between treated and control cultures are indicated by (*). Statistically significant (0.0005 ≤ *P* ≤ 0.005; n ≥ 6) differences between differentiated and undifferentiated IECs are indicated by (#). **A-B.** Results obtained with -2PC Caco-2/15 cells were highly similar to those shown here for HIEC cells.

As expected from our previous reports [29-32], the exposure to IgG’s did not affect the survival of either undifferentiated or differentiated IECs, as assessed by DNA laddering (Figure 3A). The inhibition of the β1 integrin subunit induced anoikis regardless of the state of differentiation, although DNA laddering appeared more abundant in differentiated IECs (Figure 3A). By contrast, the inhibition of β4 did not affect the survival of undifferentiated cells, whereas it induced anoikis in differentiated ones (Figure 3A).

To further confirm these results, anoikis was instead measured by ISEL, and the data from treated (blocking mAbs or generic IgG’s) cultures were then compared to those of basal (untreated) controls. Again, IgG’s did not affect the survival of undifferentiated or differentiated IECs (Figure 3B). However, the inhibition of β1 induced cell death regardless of the state of differentiation (Figure 3B). Additionally, such negative impact on cell survival was significantly greater in differentiated cells (Figure 3B), in keeping with the observed greater intensity of DNA laddering (Figure 3A). Lastly, the inhibition of β4 once again failed to affect the survival of undifferentiated IECs, while producing significant cell death in differentiated ones (Figure 3B).

**Figure 4:**
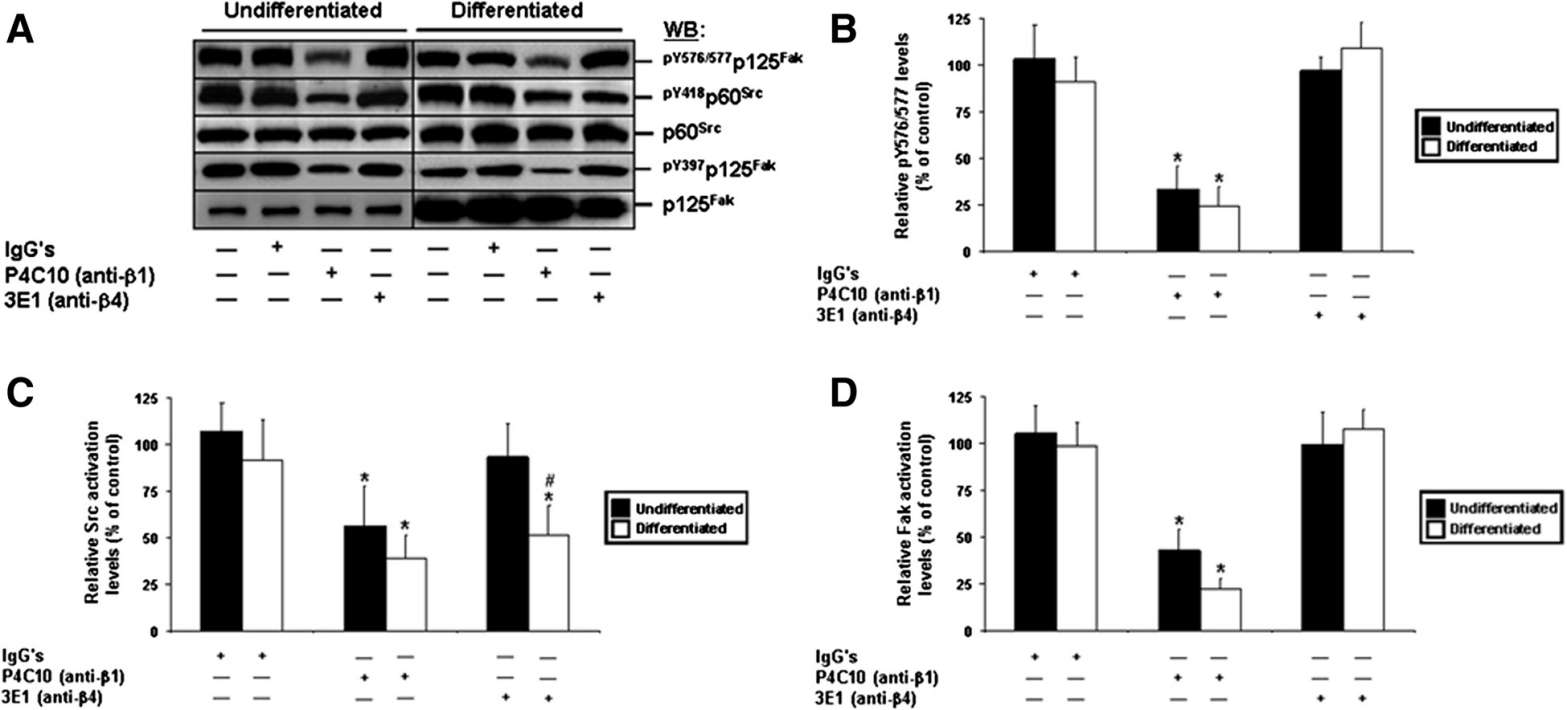
**Engagement of Fak and Src by β1 and β4 integrins in the suppression of human IEC anoikis. A.** Representative (n ≥ 4) WB analyses of the activation of Src and Fak, and verifications of Fak-Src interactions, from -2PC (*Undifferentiated*) and 30PC (*Differentiated*) Caco-2/15 adhering cell cultures maintained 24 h serum-free (control) with mouse IgG’s, P4C10 (β1 binding activity-blocking mAb), or 3E1 (β4 binding activity-blocking mAb). Specific antibodies for ^pY576/577^p125^Fak^, ^pY418^p60^Src^ and ^pY397^p125^Fak^, as well as for respective total protein forms, were used. **B-D.** -2PC (*Undifferentiated*; filled columns) and 30PC (*Differentiated*; open columns) Caco-2/15 cell cultures were maintained and processed as in **(*A*)**, except that the relative pY576/577 levels of Fak **(*B*)**, as well as the relative activation levels of Src **(*C*)** and Fak **(*D*)**, were established in comparison to controls. **A-D.** Results obtained with HIEC cells were highly similar to those shown here for -2PC Caco-2/15 cells. **B-D.** Statistically significant (0.0001 ≤ *P* ≤ 0.001; n ≥ 4) differences between treated and control cultures are indicated by (*). Statistically significant (0.0005 ≤ *P* ≤ 0.005; n ≥ 4) differences between differentiated and undifferentiated IECs are indicated by (#).

We then analyzed the relative activation levels of Fak and Src, as well as functional Fak-Src interactions, in the same treated and untreated cell cultures. As expected [29,32], the exposure to IgG’s did not affect the activation of either Fak or Src, and did not influence Fak-Src interactions (Figure 4A-D). However, the inhibition of β1 resulted in a significant down-activation of both Fak and Src, as well as in a significant decrease in Fak-Src interactions, regardless of the state of differentiation (Figure 4A-D). These effects were similar as those observed when cells were maintained in suspension (see previous section). By contrast, the inhibition of β4 did not affect the activation of Fak in undifferentiated or differentiated cells (Figure 4A, D), as expected [4,6,12,13,29]. Interestingly, the inhibition of this subunit did result in a significant down-activation of Src in differentiated IECs only (Figure 4A, C), while at the same time failing to affect functional Fak-Src interactions regardless of the state of differentiation (Figure 4A-B).

Hence, these data altogether, along with those of the previous section, indicate that β1 and β4 integrins perform distinct contributions in the suppression of IEC anoikis not only according to the state of differentiation, but also via their properties in engaging Fak and/or Src.

### Differentiation state-distinct contributions of α2, α3, α5 and α6 integrin subunits in the suppression of anoikis in human IECs

**Figure 5:**
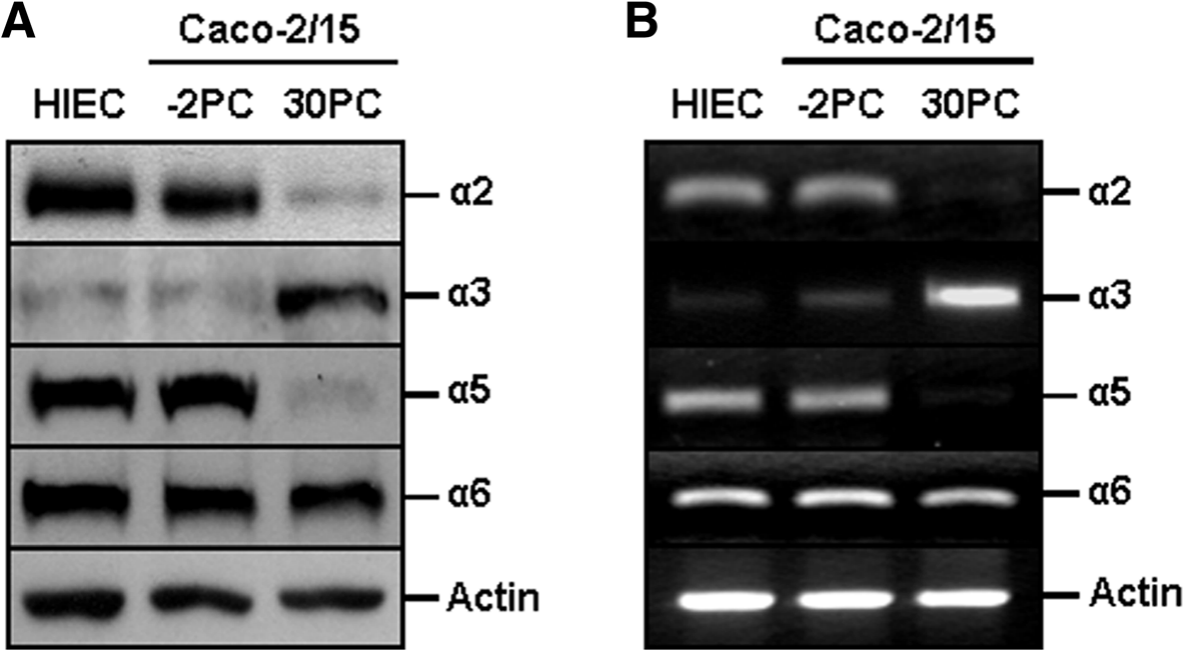
**Differentiation state-distinct expression profiles of α2, α3, α5 and α6 integrin subunits, in human IECs. A-B.** Representative (n ≥ 3) WB **(*A*)** and RT-PCR **(*B*)** analyses of the expression of the α2, α3, α5 and α6 integrin subunits, in undifferentiated (*HIEC*, *Caco-2/15 -2PC*) and differentiated (*Caco-2/15 30PC*) adhering human IEC cultures, using specific antibodies **(*A*)** or primers **(*B*)**. Actin was used as reference protein **(*A*)** and mRNA **(*B*)**.

To further understand the differentiation state-distinct contributions of the β1 and β4 integrin subunits in the suppression of anoikis, IECs were exposed to specific mAbs directed to the extracellular domains of the α2 (mAb P1E6), α3 (mAb P1B5), α5 (mAb P1D6), or α6 (mAb GoH3) integrin subunits, allowing for the inhibition of their binding activities. Again, controls constituted of cultures exposed to mouse IgG’s, or to no antibodies/IgG’s. It must be specified here that human crypt IECs express predominantly the α2 and α5 integrin subunits, with little or no α3, whereas villus cells express predominantly the α3 integrin subunit, with little or no α2 and α5 [22,23,39]. Accordingly, HIEC, undifferentiated Caco-2/15 cells, and differentiated Caco-2/15 cells, display *in vitro* expression profiles of these integrin subunits that are similar to their *in vivo* crypt and villus counterparts, respectively (Figure 5A-B). In any case, it is known that the α2, α3 and α5 integrin subunits partner with β1, in human IECs [4,22-24,26,27]. Conversely, the α6 integrin subunit is expressed in human IECs regardless of the state of differentiation (Figure 5A-B) [39-41]. However, it partners exclusively with the β4 subunit and, consequently, constitutes the α6β4^ctd-^ and α6β4 receptors in undifferentiated/crypt and differentiated/villus cells, respectively [22,23,26,39-41].

**Figure 6:**
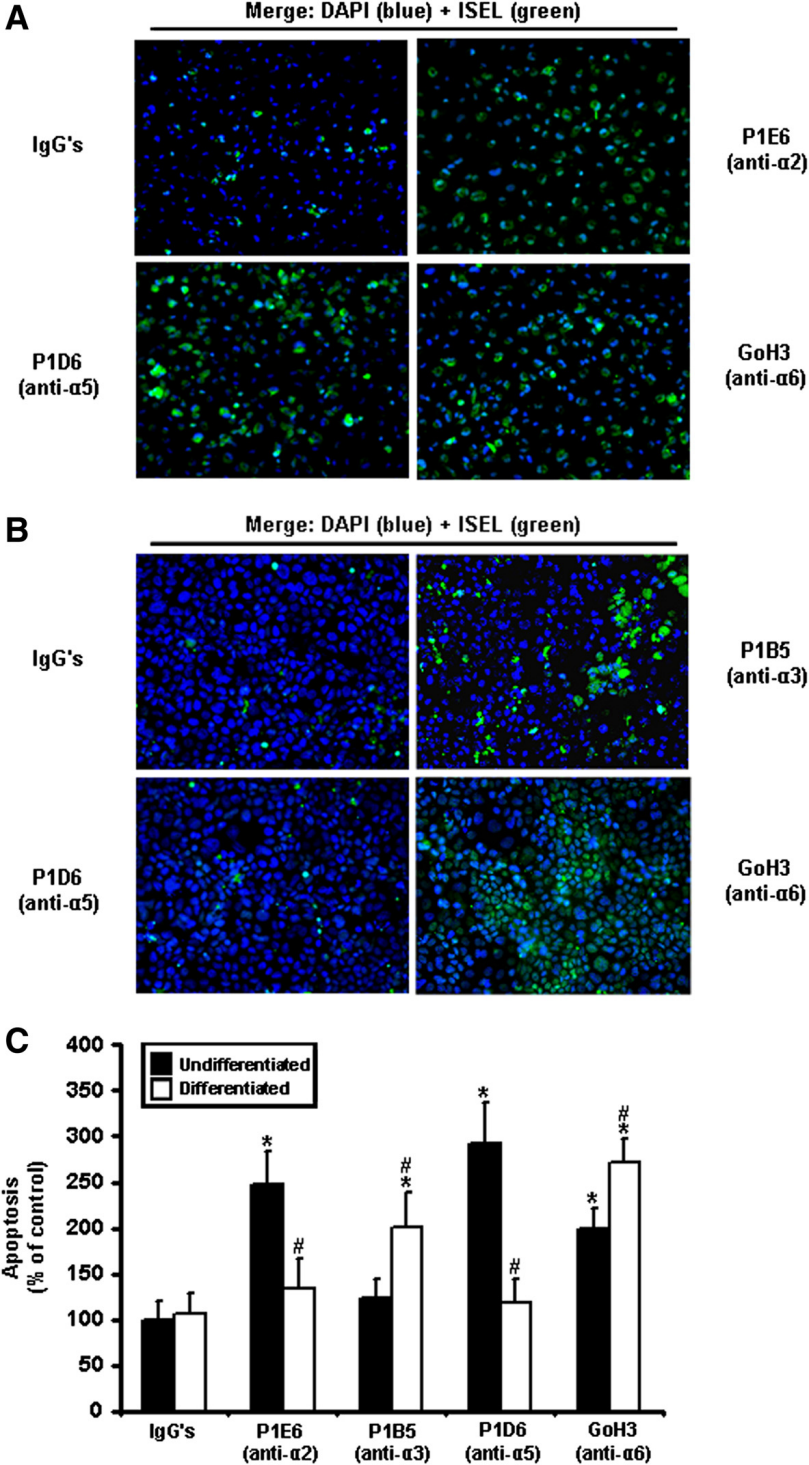
**Differentiation state-distinct contributions of α2, α3, α5 and α6 integrin subunits, in the suppression of human IEC anoikis. A.** Representative (n ≥ 3) double labeling-merged immunofluorescence micrographs of adhering HIEC cell cultures maintained 24 h serum-free (control) with mouse IgG’s, P1E6 (α2 binding activity-blocking mAb), P1D6 (α5 binding activity-blocking mAb), or GoH3 (α6 binding activity-blocking mAb). ISEL (*green*) was thereafter performed, with DAPI (*blue*) counter-staining of nuclei. **B.** Same as in **(*A*)**, except that adhering 30PC Caco-2/15 cells were maintained 24 h serum-free (control) with mouse IgG’s, P1B5 (α3 binding activity-blocking mAb), P1D6 (α5 binding activity-blocking mAb), or GoH3 (α6 binding activity-blocking mAb). **C.** Adhering HIEC (*Undifferentiated*; filled columns) and 30PC Caco-2/15 (*Differentiated*; open columns) cell cultures were maintained 24 h serum-free (control) with mouse IgG’s, P1E6, P1B5, P1D6, or GoH3. ISEL assays were performed and compared to controls. Statistically significant (0.0001 ≤ *P* ≤ 0.001; n ≥ 3) differences between treated and control cultures are indicated by (*). Statistically significant (0.0005 ≤ *P* ≤ 0.005; n ≥ 3) differences between differentiated and undifferentiated IECs are indicated by (#). **A-B.** Original magnifications: 20X. **A, C.** Results obtained with -2PC Caco-2/15 cells were highly similar to those shown here for HIEC cells.

As assessed by ISEL, the inhibition of the α2 integrin subunit induced significant cell death in undifferentiated cells, but not in differentiated ones (Figure 6A, C). Likewise, the inhibition of α5 produced significant anoikis in undifferentiated IECs only (Figure 6A-C). By contrast, the inhibition of α3 induced significant cell death in differentiated IECs, but not in undifferentiated ones (Figure 6B-C). Finally, the inhibition of α6 produced abundant anoikis regardless of the state of differentiation, although its impact to this effect was significantly greater in differentiated cells (Figure 6A-C).

Taken altogether with our previous results regarding the β1 and β4 integrin subunits, these data indicate that the α2, α3, α5, and α6 subunits perform differentiation state-selective contributions in the suppression of human IEC anoikis not only according to their differentiation state-associated expression, but also depending on which β subunit (and variant) they are known to partner with.

### Differentiation state-distinct contributions of α2, α3, and α5, in the engagement of Fak and Src by the β1 integrin subunit, in the suppression of human IEC anoikis

**Figure 7:**
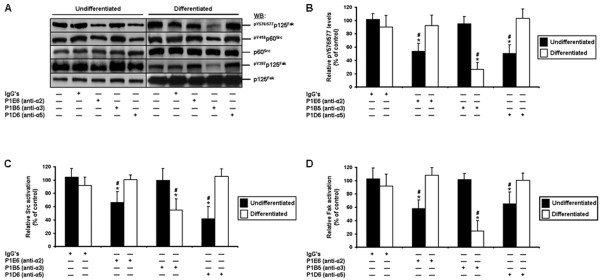
**Contributions of the α2, α3, and α5 integrin subunits in the engagement of Fak and Src, in human IECs. A.** Representative (n ≥ 3) WB analyses of Src and Fak, and verifications of Fak-Src interactions, from adhering HIEC (*Undifferentiated*) and 30PC Caco-2/15 (*Differentiated*) cell cultures maintained 24 h serum-free (control) with mouse IgG’s, P1E6 (α2 binding activity-blocking mAb), P1B5 (α3 binding activity-blocking mAb), or P1D6 (α5 binding activity-blocking mAb). Specific antibodies for ^pY576/577^p125^Fak^, ^pY418^p60^Src^ and ^pY397^p125^Fak^, as well as for respective total protein forms, were used. **B-C.** HIEC (*Undifferentiated*; filled columns) and 30PC Caco-2/15 (*Differentiated*; open columns) cell cultures were maintained and processed as in **(*A*)**, except that the relative pY576/577 levels of Fak **(*B*)**, as well as the relative activation levels of Src **(*C*)** and Fak **(*D*)**, were established in comparison to controls. **A-D.** Results obtained with -2PC Caco-2/15 cells were highly similar to those shown here for HIEC cells. **B-D.** Statistically significant (0.0001 ≤ *P* ≤ 0.001; n ≥ 3) differences between treated and control cultures are indicated by (*). Statistically significant (0.0005 ≤ *P* ≤ 0.005; n ≥ 3) differences between differentiated and undifferentiated IECs are indicated by (#).

Since the α2, α3, and α5 integrin subunits partner with β1 in human IECs [4,22-24,26,27], we analyzed the relative activation levels of Fak and Src, as well as Fak-Src interactions, following the mAb-mediated inhibition of the binding activity of each of these three α subunits. In undifferentiated cells, the inhibition of α2 and α5, but not α3, resulted in a significant down-activation of both Fak and Src, as well as in a significant decrease in Fak-Src interactions (Figure 7A-D). By contrast, in differentiated cells, the inhibition of α3, but not α2 or α5, resulted in a significant down-activation of Fak and Src, as well as in a significant decrease in Fak-Src interactions (Figure 7A-D). Overall, these results further confirm the differentiation state-distinct contributions of α2, α3 and α5 in the suppression of anoikis (Figure 6), in addition to corroborating the results obtained following the inhibition of Fak, of Src, and of the β1 subunit (Figures 2, 3, 4).

Altogether, these data indicate that the α2β1, α3β1, and α5β1 integrins perform differentiation state-selective functions in the Fak/Src signaling-mediated suppression of human IEC anoikis.

### Differentiation state-distinct contributions of α6 in the engagement of Src by the β4 integrin subunit, in the suppression of human IEC anoikis

As already noted, the α6β4^ctd-^ and α6β4 integrins are expressed in undifferentiated/crypt and differentiated/villus cells, respectively [22,23,26,39-41]. Interestingly, the α6β4^ctd-^ receptor is non-functional for anchorage, as ascertained by the inhibition of α6, β4, or both, in human IEC adhesion assays [38]. Thus, our findings in these cells that the inhibition of β4 binding activity failed to induce anoikis (Figure 3A-B), or impact significantly on Src activation (Figure 4A, C), corroborated such a lack of binding functionality for α6β4A^ctd-^. However, our additional findings that, in these same undifferentiated IECs, the inhibition of α6 binding activity nevertheless induced cell death (Figure 6A, C), constituted a contradiction to this effect.

**Figure 8:**
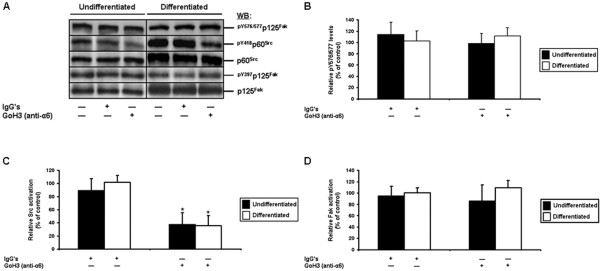
**Contributions of the α6 integrin subunit in the engagement of Fak and Src, in human IECs. A.** Representative (n ≥ 3) WB analyses of Src and Fak from adhering HIEC (*Undifferentiated*) and 30PC Caco-2/15 (*Differentiated*) cell cultures maintained 24 h serum-free (control) with mouse IgG’s, or GoH3 (α6 binding activity-blocking mAb). Specific antibodies for ^pY576/577^p125^Fak^, ^pY418^p60^Src^ and ^pY397^p125^Fak^, as well as for respective total protein forms, were used. **B-D.** HIEC (*Undifferentiated*; filled columns) and 30PC Caco-2/15 (*Differentiated*; open columns) cell cultures were maintained and processed as in **(*A*)**, except that the relative pY576/577 levels of Fak **(*B*)**, as well as the relative activation levels of Src **(*C*)** and Fak **(*D*)**, were established in comparison to controls. **A-D.** Results obtained with -2PC Caco-2/15 cells were highly similar to those shown here for HIEC cells. **B-D.** Statistically significant (0.0001 ≤ *P* ≤ 0.001; n ≥ 3) differences between treated and control cultures are indicated by (*).

We therefore analyzed the relative activation levels of Fak and Src in undifferentiated and differentiated IECs, following the inhibition of α6 binding activity. As always, controls constituted of cultures exposed to mouse IgG’s, or to no antibodies/IgG’s. In undifferentiated cells, the inhibition of α6 caused a significant down-regulation of Src (Figure 8A, C), which was in line with its induction of cell death (Figure 6A, C), but again in contradiction with the failure of the inhibition of β4 to impact on either parameters (Figure 3A-B, and Figure 4A, C). However, the inhibition of α6 did not impact significantly on functional Fak-Src interactions (Figure 8A-B), or the activation of Fak itself (Figure 8A-B, D), as similarly observed when β4 binding activity was inhibited (Figure 4A, D).

In differentiated cells, α6 inhibition likewise caused a significant down-activation of Src, while failing to affect Fak-Src interactions, or Fak activation (Figure 8A-D). However, these results in differentiated IECs were consistent with those already obtained via the inhibition of β4 (Figure 4A-D). Also, these agreed with regards to the induction of anoikis in these same differentiated IECs, when either α6 or β4 were inhibited (Figure 3A-B, Figure 6B-C).

**Figure 9:**
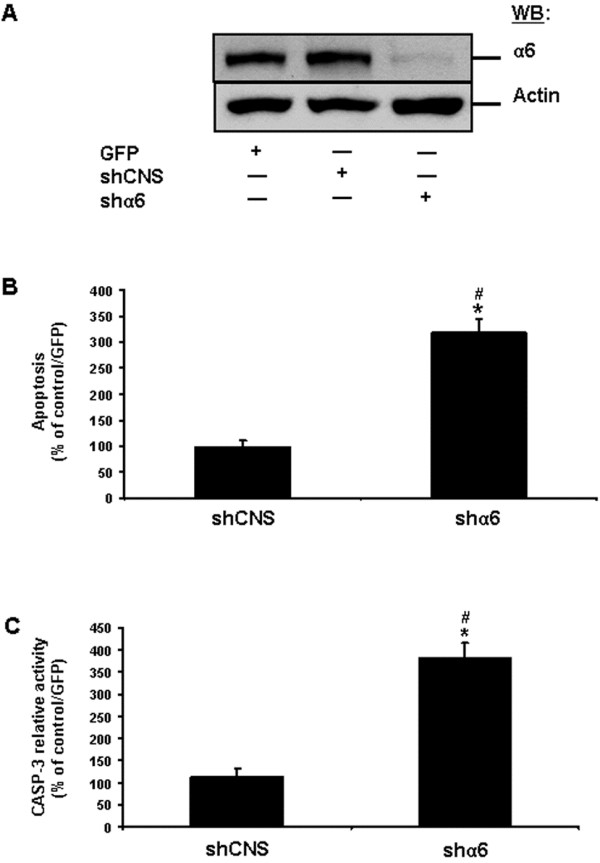
**Impact of the knockdown of the α6 integrin subunit on the survival of human IECs. A.** Representative (n ≥ 3) WB analyses of α6 and actin from adhering HIEC cell cultures, following their infection by a lentivirus carrying GFP (control), shCNS or shα6. Specific antibodies for α6 and actin were used. **B.** Adhering HIEC cell cultures were infected as in **(*A*)**, except that ISEL assays were performed and compared to controls. **C.** Adhering HIEC cell cultures were infected as in **(*A*)**, except that CASP-3 relative activity was established, by comparison to controls. **A-C.** Results obtained with -2PC Caco-2/15 cells were highly similar to those shown here for HIEC cells. **B-C.** Statistically significant (0.0001 ≤ *P* ≤ 0.001; n ≥ 3) differences between treated and control cultures are indicated by (*). Statistically significant (0.0005 ≤ *P* ≤ 0.005; n ≥ 3) differences between shα6 and shCNS are indicated by (#).

In an attempt to resolve the apparently enduring inconsistencies in our results pertaining to each subunit of α6β4^ctd-^ in undifferentiated IECs, we opted for the shRNA-mediated knockdown of α6 in these cells, using a lentiviral approach of delivery. As shown in Figure 9A, the shα6 we used caused a reduction in the expression of the α6 subunit by at least 80%, as compared to when cells were infected with a shCNS, or with GFP. We then verified the impact of such α6 knockdown on the survival of undifferentiated IECs. As expected from our α6 inhibition studies (Figure 6A, C), the shα6 induced significant anoikis in undifferentiated human IECs (Figure 9B-C).

**Figure 10:**
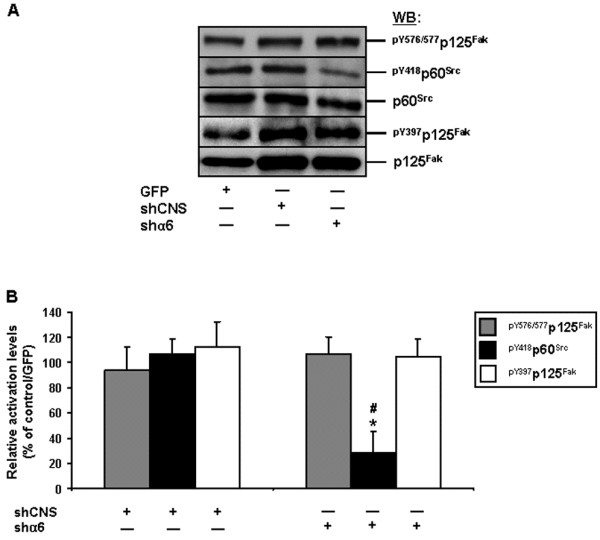
**Impact of the knockdown of the α6 integrin subunit on Fak and Src activation, in human IECs. A.** Representative (n ≥ 3) WB analyses of Src and Fak, and verifications of Fak-Src interactions, from adhering HIEC cell cultures following their infection by a lentivirus carrying GFP (control), shCNS or shα6. Specific antibodies for ^pY576/577^p125^Fak^, ^pY418^p60^Src^ and ^pY397^p125^Fak^, as well as for respective total protein forms, were used. **B.** Adhering HIEC cell cultures were infected and processed as in **(*A*)**, except that the relative pY576/577 levels of Fak (grey columns), as well as the relative activation levels of Src (filled columns) and Fak (open columns), were established in comparison to controls. Statistically significant (0.0001 ≤ *P* ≤ 0.001; n ≥ 3) differences between treated and control cultures are indicated by (*). Statistically significant (0.0005 ≤ *P* ≤ 0.005; n ≥ 3) differences between shα6 and shCNS are indicated by (#). **A-B.** Results obtained with -2PC Caco-2/15 cells were highly similar to those shown here for HIEC cells.

We also analyzed the relative activation levels of Fak and Src, as well as Fak-Src interactions, following such knockdown of α6. As expected from our α6 inhibition studies (Figure 8A-B, D), the shα6 caused a significant reduction of Src activation without impacting significantly on the activation of Fak, or Fak-Src interactions (Figure 10).

Therefore, these data altogether indicate that both α6β4^ctd-^ and α6β4 contribute in the suppression of anoikis via the engagement of a functional pool of Src that does not interact with Fak and, therefore, which is distinct from the one engaged by β1 integrins. However, these results also suggest that the contributions of α6β4^ctd-^ in the suppression of anoikis in undifferentiated IECs appear to be primarily dependent on its α6 subunit, whereas α6β4 enacts significantly greater contributions than it’s anchorage non-functional counterpart in the suppression of anoikis in differentiated IECs, in addition to doing so through a dependence on both its α6 and β4 subunits.

## Discussion

**Figure 11:**
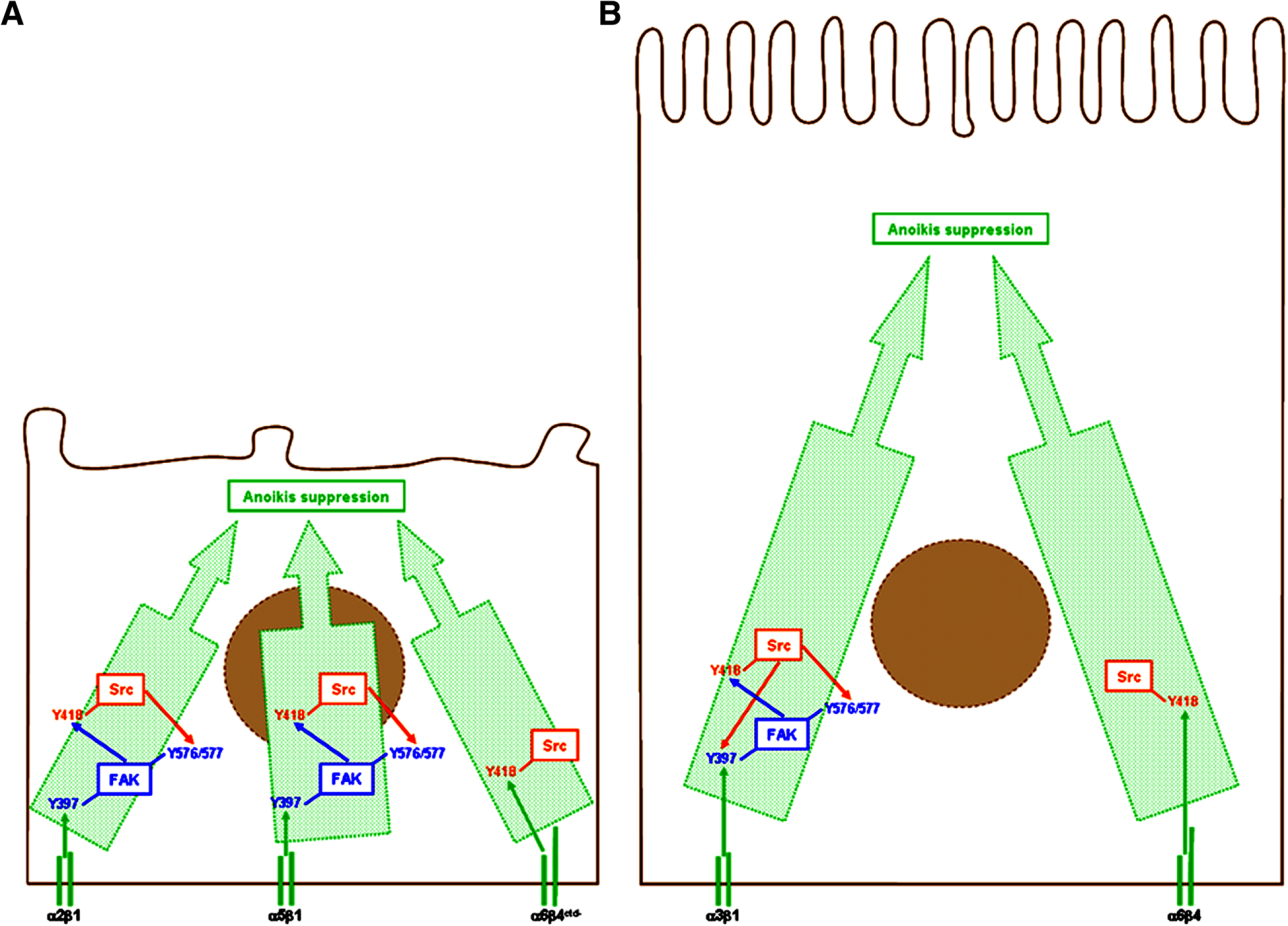
**Differentiation state-selective contributions of α2β1, α3β1, α5β1, and α6β4 integrins in the suppression of anoikis in human IECs.** Schematic drawing of an undifferentiated IEC **(*A*)** and its differentiated counterpart **(*B*)**, illustrating how α2β1, α3β1, α5β1, and α6β4 integrins contribute in the engagement of Fak/Src signaling for the suppression of anoikis. **A.** In undifferentiated IECs, α2β1 and α5β1 (but not α3β1) contribute in the engagement and activation of Fak (assessed by Y397 residue phosphorylation) via the classical requirement of both α and β subunits for integrin functionality and signalling. Fak then engages and activates Src (assessed by Y418 residue phosphorylation) which, in turn, enacts functional Fak-Src interactions (assessed by phosphorylation of Y576/577 residues of Fak). In parallel, α6β4^ctd-^ engages and activates a distinct pool of Src that does not interact functionally with Fak. Furthermore, such Src engagement by α6β4^ctd-^ is enacted in a primarily α6 subunit-dependent manner. **B.** In differentiated IECs, α3β1 (but not α2β1 or α5β1) engages and activates Fak, again via the classical requirement of both α and β subunits. Fak then engages and activates Src which, in turn, enacts functional Fak-Src interactions. However, in this context, Src contributes reciprocally to Fak activation. In parallel, α6β4 engages and activates a distinct pool of Src that does not interact functionally with Fak. Additionally, the engagement of Src by α6β4 is now enacted via the classical requirement of both α and β subunits. **A-B.** Such integrin subunit- and differentiation state-selective roles of α2β1, α3β1, α5β1, and α6β4 integrins, in the engagement of Fak and/or Src, are likely to contribute to the outcome of differentiated IECs being more sensitive to anoikis than their undifferentiated counterparts.

In the present study, we investigated the differentiation state-specific roles of the α2, α3, α5, α6, β1, and β4 integrin subunits in the suppression of IEC anoikis, including with regards to their contributions in the activation of Fak and/or Src. Human undifferentiated/crypt and differentiated/villus IECs express distinct repertoires of integrins (and variants) [4,22-24,26,38-41]. Particularly, undifferentiated IECs predominantly express α2β1, α5β1 and α6β4^ctd-^, whereas differentiated ones predominantly express α3β1 and α6β4 [4,22-24,26,38-41]. Herein, we report that differentiated IECs exhibit a greater sensitivity to anoikis than undifferentiated ones. This implicates an earlier onset of anoikis when kept in suspension, as well as significantly greater contributions from β1 and β4 integrins in the suppression of anoikis in differentiated cells, and functional distinctions between β1 and β4 integrins in engaging both Fak and Src, or Src only, respectively. Accordingly, Fak performs significantly greater contributions in the suppression of anoikis in differentiated cells. We also show that α2β1 and α5β1 suppress anoikis in undifferentiated cells, whereas α3β1 does so in differentiated ones (Figure 11). Furthermore, we provide evidence that α6β4^ctd-^, which is expressed in undifferentiated IECs and is non-functional for anchorage [26,38-41], contributes nevertheless to the suppression of anoikis in a primarily α6 subunit-dependent manner. Additionally, we show that α6β4, which is expressed in differentiated cells and is anchorage-functional [26,38-41], not only performs significantly greater contributions than its anchorage non-functional counterpart in the suppression of anoikis, but does so through a dependence on both of its subunits (Figure 11). Hence, the suppression of human IEC anoikis implicates differentiation state-selective repertoires of integrins, which in turn results into distinctions in anoikis regulation, and sensitivity, between undifferentiated and differentiated IECs.

Our present study, coupled with our previous observations regarding human IEC survival and anoikis [29-37], now firmly establish that differentiated/villus cells are more sensitive to anoikis than their undifferentiated/crypt counterparts. Such differentiation state-associated distinctions likely constitute a major physiological underpinning for the process of exfoliation-by-anoikis of obsolete IECs, upon them reaching the apex of villi [4,23,24,27,28,42]. This could also account for the observations that mechanical/shearing forces in the intestine occasionally cause incidental anoikis in villus cells only [42,43]. To this effect, our findings herein that (α2, α3, α5)β1 and α6β4 integrins selectively contribute to the suppression of IEC anoikis according to the state of differentiation, in correlation with the differential expression profiles of these same integrins (and variants) exhibited by crypt and villus cells [4,22-24,26,27,39], identify integrins as the main functional determinants for such differentiation state-associated sensitivity to anoikis. This is further supported by the observations that differentiated IECs are more sensitive to the inhibition (and/or knockdown) of β1, α6, and β4 (this study; [29-32]). Additionally, integrin-mediated cell polarization and actin organization confer greater sensitivity to anoikis [1,3,4,9,12,17-20,44,45], and differentiated/villus IECs are highly polarized in sharp contrast to their poorly polarized undifferentiated/crypt counterparts [23,39,46-48]. In a similar vein, α6β4-mediated hemidesmosome formation and intermediate filament organization likewise confer greater anoikis sensitivity [1,3,4,6,9,12,13,44],[45], whereby the anchorage functional α6β4-expressing villus cells bear type II hemidesmosomes, and the anchorage non-functional α6β4^ctd-^-expressing crypt cells lack hemidesmosomes altogether [22,26,38,46,47]. However, it is noteworthy that some integrins may not suppress anoikis, as one would expect [1-4,9,16,17,20,27], but rather sensitize cells to the process [4,19,20]. For instance, it was shown recently that α8β1 is expressed in undifferentiated/crypt IECs (but not in differentiated/villus ones), and that the knockdown of the α8 subunit not only fails to impact negatively on the survival of these cells, but actually confers a measure of anoikis resistance to them [49]. Thus, much remains to be understood of the regulatory determinants that confer differentiation state-associated distinctions in anoikis suppression and sensitivity, including individual integrins themselves.

It is recognized that cell survival and death implicate regulatory determinants that are distinct according not only to the species and tissue, but also to the state of cell differentiation [4,15,24,44]. So far, this concept has been largely verified in the human intestinal epithelium [4,23,24,27,28,50,51]. For instance, IECs exhibit differentiation state-distinct expression profiles of anti- and pro-apoptotic Bcl-2 homologs, said profiles being established during the differentiation process [29,50,52,53]. Additionally, the expression of individual Bcl-2 homologs is subjected to regulatory mechanisms that are specific according to the differentiation status of IECs [29,34,37]. Such concept of differentiation state-distinct modulations of cell survival and apoptosis inherently suggests that the regulation of anoikis likewise implicates differentiation state-specific determinants [4,24]. In the particular case of human IECs, several lines of evidence now support this. As already highlighted above, human IECs display differentiation state-related distinctions in anoikis sensitivity, and this is associated with a differentiation state-selective implication of integrins in the regulation of anoikis. To that effect, the expression profiles of individual Bcl-2 homologs are affected distinctively according to the state of differentiation, following induction of anoikis by maintaining IECs in suspension [29,34,37]. Additionally, differentiated cells exhibit lower relative levels of activated Fak than undifferentiated ones and, consequently, are more sensitive to its inhibition (this study; [29-33,37]). In this respect, Fak impacts in a differentiation state-distinct manner on the expression of individual Bcl-2 homologs [29,34,37]. Although the relative levels of activated Src are similar between undifferentiated and differentiated cells, and consequently show similar sensitivities to its inhibition (this study), Src nonetheless impacts distinctively on the individual expression of Bcl-2 homologs according to the state of differentiation [4,32-34]. Furthermore, we have previously reported that although the integrin/Fak/Src engagement of the MEK/Erk pathway is primarily Src-dependent regardless of the state of human IEC differentiation, this same pathway plays a marginal role (at best) in the suppression of anoikis in undifferentiated cells, as opposed to its substantial roles to that effect in differentiated ones [29,30,33,34,37]. Similarly, isoforms of the PI3-K/Akt pathway perform differentiation state-distinct roles in the integrin/Fak/Src-mediated regulation of anoikis, in relation to their selective engagement by Fak, or Src, and again according to the differentiation status of human IECs [29-35,37]. Lastly, we have previously demonstrated that the integrin/Fak/Src-mediated suppression of anoikis in human IECs includes the inhibition of the pro-apoptotic activation of p38β^SAPK^ and p38δ^SAPK^ in undifferentiated and differentiated cells, respectively [30,34,37]. Hence, taking into account that similar lines of evidence have been mounting from studies in other cell types [1,4,9,12,13,19,44,45], it is now clear that the integrin-mediated control of anoikis is not only tissue type- and species context-dependent, but also differentiation state-selective.

In this respect, our observations that the inhibition of Src does not impact on the activation of Fak in undifferentiated cells while resulting in Fak’s down-activation in differentiated ones (this study), coupled to those that show a dependence on Fak for Src’s activation regardless of the state of differentiation (this study; [32-35]), are strongly suggestive of a differentiation state-distinct crosstalk between Fak and Src with regards to their respective activation (Figure 11). Signaling by β1 integrins is typically initiated by the recruitment and activation of Fak at the cytoplasmic tail of the β subunit. In turn, Fak recruits and activates Src [1,2,4,8-12,16,17,20,54],[55]. Conversely, Src may be first to be engaged, and then recruit/activate Fak [4,11,54,55]. Although recent studies point to the identity of individual integrins specifically involved, as well to the intervention of integrin-associated cytoplasmic molecules such as paxillin, talin or filamin, much remains to be understood of the determinants that dictate the order of engagement of Fak and Src, as well as in defining their mutual dependence (or not) for their activation [54,55].

On a related note, it is intriguing that the inhibition of β1 integrins leads to the down-activation of Fak and Src, along with a consequent drop in functional Fak-Src interactions, whereas the inhibition of α6 and/or β4 down-activates Src without affecting Fak, or Fak-Src interactions, regardless of the state of IEC differentiation (this study; [32,34,35]). While these observations fall in line with the current understanding that α6β4-mediated signaling engages Src, whereas that of β1 integrins engages both Fak and Src [1,2,4,8-13,16,17,20,26],[54,55], these nonetheless suggest the existence of separate, functional pools of Src in human IECs – namely one engaged by β1 integrins/Fak, and the other engaged by α6β4^ctd-^/α6β4 (Figure 11). The existence of spatially and functionally compartmentalized intracellular pools of signaling molecules is now well recognized [4,8,11,56,57]. Incidentally, two other separate pools of Src, likewise involved in the regulation of cell survival and/or anoikis, have been previously identified in IECs: one associated with E-cadherin junctional complexes [44,56,57], and one associated with RTK signaling [33]. In the case of the former, it remains to be fully understood how E-cadherin-mediated Src signaling may crosstalk with that of integrins (with Fak or not) [45,56,57]. In the case of the latter, it is already established that extensive crosstalk occurs between integrins and RTKs in the regulation of virtually all known cell processes, often through Src [4,8,11,20,33,44,45,58]. Therefore, further studies are warranted in order to better understand the underpinnings of the crosstalk between Fak and distinct functional pools of Src, particularly with regards to the roles of such crosstalk in the regulation of anoikis.

Another finding in the present study is that the α6β4 integrin expressed in undifferentiated IECs, which is non-functional for anchorage and/or hemidesmosome formation [26,38,40,41,47], contributes nevertheless to the suppression of anoikis and does so primarily in an α6-dependent manner (Figure 11). By contrast, the α6β4 integrin expressed in differentiated IECs not only enacts significantly greater contributions than its anchorage non-functional counterpart in the suppression of anoikis, but does so through a dependence on its two subunits (Figure 11). Considering that this integrin is functional for both anchorage and hemidesmosome formation [26,38,40,41,47], it is therefore not surprising that differentiated IECs are more sensitive to its inhibition (whether via α6 or β4), or that its allosteric activation corresponds to the classical requirement of both α and β subunits for integrin functionality and signaling [5,7,8]. Incidentally, human IECs express the mRNA-splicing cytoplasmic domain-variants α6A and α6B, whereby undifferentiated/crypt cells express α6Aβ4^ctd-^ and differentiated/villus cells express α6Bβ4 [26,38,40,41]. To that effect, α6Aβ4^ctd-^ has been shown to promote proliferation in undifferentiated/crypt IECs [40,41]. Hence, our observations that α6(A)β4^ctd-^ engages Src, and thus contributes to anoikis suppression in undifferentiated IECs, constitutes an additional instance of cell process-implication capacity for this integrin variant. Interestingly, the forced over-expression of α6B in undifferentiated IECs leads to their growth arrest [40,41]. Considering our observations regarding the α6(A)-dependence for α6(A)β4^ctd-^ in engaging Src and suppressing anoikis in undifferentiated cells, as well as the emerging evidence of signaling abilities by the α6 integrin subunit [13,26,59], these data altogether raise further the question of the variant-specific roles for α6A and α6B in IEC processes – including in the suppression of anoikis.

Lastly, although our use of binding activity-blocking antibodies allowed for the discrimination of the impacts of the inhibition of a given integrin subunit between undifferentiated and differentiated IECs, these same biological tools did not allow for a similar discrimination between the individual integrin subunits analyzed. This is simply due to the inherent variability of efficiency between the specific antibodies used. As examples, while we were able to conclude with confidence that the inhibition of α2β1 contributes to anoikis suppression in undifferentiated cells only, it remains inconclusive whether β1 integrins perform the greater roles in anoikis suppression than β4 ones, or that α5β1 is more important than α2β1 in the suppression of anoikis in undifferentiated cells.

## Conclusions

This study has provided evidence that distinctions in anoikis susceptibility, between undifferentiated/crypt and differentiated/villus IECs, implicate differentiation state-selective repertoires of (α)β1 and α6β4 integrins (and variants), which result into differentiation state-distinctions in the overall regulation of human IEC anoikis (Figure 11). In this respect, the present findings altogether provide further functional understanding of the concept that cell survival and suppression of anoikis are subjected to differentiation state-selective mechanisms. Additionally, we specifically identify the α2β1, α5β1, and α6β4^ctd-^ integrins as determinants of anoikis regulation in undifferentiated IECs, as well as α3β1 and α6β4 as comparable determinants in differentiated ones. However, these findings in no way exclude similar implications for other integrins (and variants) that are expressed by human IECs [22,23,26,39-41]. Further studies, along with the present findings, should provide a greater understanding of the inherent complexities of the integrin-mediated modulation of anoikis not only within the context of normal tissue homeostasis, but also within the physiopathological context of tissue dysfunction.

## Methods

### Materials

Specific antibodies directed against integrin subunits α2, α3, α5 and α6, as well as against p125^Fak^, the phosphotyrosine397 activated form of p125^Fak^ (^pY397^p125^Fak^), the Src-phosphorylated tyrosine 576 and 577 residues of p125^Fak^ (^pY576/577^p125^Fak^), p60^Src^, the phosphotyrosine418 activated form of p60^Src^ (^pY418^p60^Src^), and actin, were used as described previously [29-38,40,41] and purchased from Abcam (Cambridge, CA), Cell Signaling Technology (Beverly, MA) and/or Millipore (Etobicoke, ON, Canada). Other materials and reagents were purchased from Sigma (Oakville, ON, Canada) and/or Fischer Scientific (St-Laurent, QC, Canada), except where otherwise specified.

### Cell culture

Two established human IEC models, which are directly relevant to human intestinal physiology and which allow the accounting of the context of crypt *Vs*. villus IEC differentiation status [26,29-35,37-41,46,48,49], were used in the present study. The human intestinal epithelial crypt (HIEC) cells are undifferentiated IECs that exhibit the morphological and functional properties of *in vivo* proliferative/undifferentiated human crypt IECs [46,48]. Although HIEC cells undergo contact-growth inhibition upon reaching confluence, they remain undifferentiated [46,48]. Cells of the Caco-2/15 line undergo a full morphological and functional differentiation process as a monolayer, which takes place spontaneously once confluence (0 days postconfluence; 0PC) has been reached, and which is completed after 25–30 days [46]. Caco-2/15 cells were used herein either as subconfluent/undifferentiated (-2PC) or postconfluent/fully differentiated (30PC) cultures. HIEC, Caco-2/15 -2PC cells and Caco-2/15 30PC monolayers express integrin subunit repertoires, and deposit ECM constituents *in vitro*, that correspond to those observed *in vivo* for crypt and villus cells, respectively (Figure 5) [26,38-41,46,48,49]. HIEC and Caco-2/15 cells were routinely cultured as we described previously [29-35,37,38,40,41,49].

### Integrin subunit binding activity-blocking assays

We used an established approach of integrin binding activity inhibition in adhering cell cultures, using integrin subunit-specific blocking antibodies (e.g. [29-32,34,35,37,60]. This approach is further facilitated by the physiological property of IECs to transcytose Igs from their apical extracellular environment to their basolateral one [23,25], thus enabling efficient access of blocking antibodies to their targeted basal, already binding on naturally-deposited ECM, integrin subunits [29-32,34,35,37]. Specific mouse monoclonal antibodies used for blocking the binding activity of integrin subunits were the following: P1E6 (Millipore), which blocks α2; P1B5 (Millipore), which blocks α3; P1D6 (Abcam), which blocks α5; GoH3 (Cell Signaling), which blocks α6 (A and/or B variants); P4C10 (Millipore), which blocks β1; and 3E1 (Millipore), which blocks β4. These antibodies have been extensively characterized/used in numerous previous studies and are efficient at blocking/inactivating their targeted integrin subunits, even when already binding (e.g. [29-32,34,35,37,38,40,41,60]). As we previously described [29-32,34,35,37], cell cultures were maintained 24 h serum-free with 100 μg/ml of either one of the blocking antibodies. Working concentrations of the antibodies used were determined previously with dose-response assays (not shown; e.g. [29-32,34,35,37,60]). Non-treated cultures were considered as basal controls, whereas cultures exposed instead to 100 μg/ml mouse IgG’s (Sigma) represented controls for the blocking antibodies, especially with regards to potential non-specific, steric encumbrance/perturbation of already binding integrins [29-32,34,35,37,38,40,41,60].

### Pharmacological inhibition of Fak, Src activity assays

Cell cultures were maintained 24 h in medium without serum (controls) or with *i*) 1 μM PF573228 (Tocris Bioscience), for the specific inhibition of Fak; or *ii*) 20 μM PP2 (Calbiochem), for the inhibition of Src. The working concentrations of the inhibitors used were determined previously with dose–response assays (not shown; [32-35]). It is noteworthy that control cultures included exposure to the same solvent as that used for inhibitors and showed no significant differences with cultures maintained in serum-free medium only (not shown; [32-35]).

### Anoikis assays

Anoikis was induced by keeping cells in suspension 0-24 h, in serum-free medium. This was done by either seeding freshly trypsinized undifferentiated cells onto poly-2-hydroxyethyl methacrylate (polyHEMA)–coated dishes, or by detaching intact monolayers of differentiated cells by gentle flushing underneath the monolayer with serum-free medium, as we previously described [29-35,37].

### CAD-mediated DNA laddering assays

DNA was isolated and the visualization of anoikis-associated CAD-mediated internucleosomal DNA fragmentation (“DNA laddering”; [2, 4, 74]), on 2% agarose gels (20 μg DNA/lane), was performed as we described elsewhere [29-35,37]. Note that the method used for DNA extraction employs Triton rather than SDS, thus often leaving behind most intact genomic DNA [29-35,37].

### Reverse transcriptase-polymerase chain reaction (RT-PCR)

Total RNA extraction and subsequent RT-PCR were carried out as described previously [30,31,35]. Specific primers for the amplification of α2, α3, α5, α6 and actin were purchased from Invitrogen Life Techologies (Grand Island, NY). Controls for reactions were: *a*) DNA without adding primers; and *b*) primers without adding DNA (not shown) [30,31,35].

### Caspase activity assays

Fluorometric caspase activity assays were performed as we previously described [35]. Specific fluorogenic substrates used were: benzyloxycarbonyl-Ile-Glu-Thr-Asp 7-amino-4-trifluoromethylcouramin (Z-IETD-AFC; Calbiochem, San Diego, CA), for CASP-8; acetyl-Leu-Glu-His-Asp 7-amino-4-trifuoromethylcouramin (Ac-LEHD-AFC; Calbiochem), for CASP-9; or acetyl-Asp-Glu-Val-Asp 7-amino-4-methylcoumarin (Ac-DEVD-AMC; Calbiochem), for CASP-3. For time-course kinetics, assays for each caspase were performed from same cultures for each respective time-point. Reactions were read with a Hitachi S-2500 Spectrofluorometer, and caspase activity was expressed as arbitrary units (AU) [35]. In other experiments, AU values of treated cultures were compared to those of non-treated/control ones, X100, in order to establish the “caspase relative activity” (expressed as “% of control”) [35].

### ISEL assays

Coverslip-grown cell cultures were processed and *in situ* terminal deoxynucleotidyl transferase (TDT)-mediated dUTP nick-end labeling (ISEL) was carried out, as we previously described [29-37]. Evaluation of ISEL-positive cells counterstained with 4′,6-diamidino-2-phenylindole (DAPI) was performed as described elsewhere [29-35,37]. Typically, apoptotic indices were compared to those of control cultures, X100 (expressed as “% of control”).

### Western blotting (WB) and relative kinase activation assays

Cell cultures were lysed in sample buffer (2.3% SDS, 10% glycerol, and 0.001% bromophenol blue in 62.5 mM Tris–HCl (pH 6.8) containing 5% β-mercaptoethanol) and processed as we described previously [29-37]. Proteins were resolved by SDS-PAGE (50 μg proteins/lane), electrotransferred, and probed as we already described [29-37]. Relative kinase activation analyses were performed as described previously [28-33,39]. Typically, immunoreactive bands were semi-quantified with Scion Image (Scion) and the relative activated levels of kinases were established with the ratios phosphorylated kinase/total kinase, which in turn were compared to control cultures, X100 (expressed as “% of control”).

### sh (small hairpin) RNA-mediated expression silencing assays

A commercially validated α6 (“shα6”, which silences the expression of both the α6A and α6B variants; Sigma) shRNA, as well as a CNS (control non-silencing; “shCNS”; [49,61]) shRNA, were cloned into the lentiviral expression vector pLentiNeoH1 (Invitrogen, Burlington, ON). A cDNA coding for Green Fluorescent Protein (GFP), cloned into the lentiviral expression vector pLentiCMV, was purchased from Origene (Rockville, MD) and used as indicator of infection efficiency. Lentivirus production and harvesting was performed with HEK (Human Embryonic Kidney) 293T cells, using the ViraPower Lentiviral packaging system (Invitrogen) according to the manufacturer’s instructions. For lentiviral infections, subconfluent (HIEC, Caco-2/15) cells were incubated 48 h (37°C) with lentivirus suspensions containing 4 μg/ml Polybrene (bromure hexadimethrin; Sigma). Infected cultures were thereafter rinsed with serum-free medium and further maintained 24 h serum-free, before processing for analyses.

### Data processing

Results and values shown represent mean ± SEM for at least three (n ≥ 3) separate experiments and/or cultures. Statistically significant differences were determined by the Student *t* test, with SigmaSTAT (Systat Software, San Jose, CA). Data were compiled, analyzed and processed with Excel (Microsoft, Redmond, WA). Except otherwise specified, images from blots, gels and scans were processed with Vistascan (Umax Technologies, Fremont, CA), Photoshop (Adobe, San Jose, CA) and PowerPoint (Microsoft).

## Abbreviations

CAD: Caspase-activated DNAse; CASP: Caspase; CNS: Control non-silencing; ECM: Extracellular matrix; Fak: Focal adhesion kinase; GFP: Green fluorescent protein; HIEC: Human intestinal epithelial crypt; IEC: Intestinal epithelial cell; ISEL: *In situ* terminal deoxynucleotidyl transferase (TDT)-mediated dUTP nick-end labeling; PC: Post-confluence; polyHEMA: Poly-2-hydroxyethyl methacrylate; RTK: Receptor tyrosine kinase; RT-PCR: Reverse transcriptase-polymerase chain reaction; SAPK: Stress-activated protein kinase; shRNA: Small harpin RNA; Src: Sarcoma tyrosine-protein kinase; WB: Western blot.

## Competing interests

The authors declare that they have no competing interests.

## Authors’ contributions

MB and ST contributed equally. MB, ST and PHV conceived hypotheses and experimental designs. MB, ST, MJD, VB and RG performed the experiments. Analysis and interpretation of data was done by MB, ST and PHV. PHV wrote the manuscript, with critical edits and significant intellectual input by JFB, and further input by MB. Additional biological tools were supplied by JFB. All authors read and approved the final manuscript.
